# Host Diversity and Phylogenetic Evolution of Phytoplasmas on Hainan Island in China, Bringing Challenges to Monitoring and Prevention of Related Plant Diseases

**DOI:** 10.3390/plants15121787

**Published:** 2026-06-10

**Authors:** Shao-Shuai Yu, Feng-Yu Yu, De-Jie Yang, Zhao-Wei Lin, Sheng-Jie Wang, Hai-Yan Che

**Affiliations:** 1 Coconut Research Institute, Chinese Academy of Tropical Agricultural Sciences, Wenchang 571339, China; 2 Key Laboratory of National Forestry and Grassland Administration on Silviculture and Forest Management in South China, Research Institute of Tropical Forestry, Chinese Academy of Forestry, Guangzhou 510520, China; 3 Environment and Plant Protection Institute, Chinese Academy of Tropical Agricultural Sciences, Haikou 571101, China

**Keywords:** *Candidatus* phytoplasma species, genetic diversity, evolutionary classification, tropical regions, epidemic monitoring, disease prevention and control management

## Abstract

Phytoplasmas are a group of wall-less, unculturable prokaryotic pathogenic bacteria that colonize the phloem of plants and are transmitted by piercing–sucking insects. As a typical tropical island province in China, Hainan Island has abundant biodiversity due to its unique geographical location and climatic conditions, which provide a favorable ecological environment for the survival and propagation of phytoplasmas, which infect different hosts, cause different symptoms, or belong to different subgroups. Based on host species, disease symptoms and 16Sr subgroups, 69 representative phytoplasma records from four 16Sr groups have been identified on Hainan Island, showing rich diversity in host range and pathogen species. The diversity of plant hosts and the evolutionary relationship of phytoplasmas not only affect the occurrence and prevalence of phytoplasma-related diseases but also bring great challenges to the epidemic monitoring, detection, diagnosis and prevention management of these diseases. This review summarizes current research progress on host diversity, phylogenetic evolution, mixed infection, diversity of transmission vectors, and geographical isolation differentiation, as well as adaptive evolution of phytoplasmas on Hainan Island. Furthermore, the challenges brought by plant host diversity and phylogenetic evolution to disease monitoring, diagnosis and prevention management are discussed. This review aims to provide a comprehensive theoretical basis for the in-depth study of phytoplasma-related diseases on Hainan Island, and to offer practical guidance for scientific monitoring, early warning and comprehensive prevention and control of these diseases.

## 1. Introduction

Phytoplasmas are a group of wall-less prokaryotic pathogens that colonize the phloem sieve elements of plants, cannot be cultured in vitro and are transmitted primarily by phloem-feeding insects [1,2]. These pathogens are responsible for a wide range of plant diseases worldwide, causing severe economic losses in agricultural, horticultural, and forestry ecosystems by inducing diverse symptoms including yellowing, witches’ broom, phyllody, stunting, and even plant death [2]. With a host range covering over 1000 plant species across more than 100 families, phytoplasmas exhibit remarkable diversity in their host adaptation and evolutionary characteristics [2,3,4]. The phylogenetic evolution of phytoplasmas, coupled with the diversity of their plant hosts and transmission vectors, poses significant challenges to the accurate detection, epidemic monitoring, and effective prevention and control of phytoplasma-associated diseases.

As a typical tropical island province in China, Hainan Island has a unique tropical monsoon climate, abundant rainfall, and high temperature year-round. The ecological environment supports extraordinary biodiversity, including a large number of tropical economic crops, ornamental plants, and wild plants. As one of the most important tropical agricultural bases in China, Hainan Island is widely planted with cash crops such as areca palm (*Areca catechu*), sugarcane (*Saccharum officinarum*), citrus (*Citrus* spp.), and pepper (*Piper nigrum*), which are crucial to the local economy and farmers’ livelihoods [5,6,7,8]. However, the rich plant diversity and suitable climatic conditions also provide an ideal habitat for the survival and spread of phytoplasmas, leading to frequent outbreaks of phytoplasma diseases in the region.

Based on the host species, disease symptoms and 16Sr subgroups, phytoplasma strains infecting different host plants, causing different symptoms or belonging to different 16Sr subgroup were employed; some strains infecting the same host plants, causing the same symptoms and belonging to the same16Sr subgroup were not included in the review. Currently, 69 representative phytoplasma records have been reported on Hainan Island, and the phytoplasmas have been classified into four major 16Sr groups, namely 16SrI, 16SrII, 16SrV, and 16SrXXXII [5,6,7]. Among these strains, the 16SrI and 16SrII groups are the dominant strains, infecting a wide range of host plants including important tropical economic crops such as areca palm, date palm (*Phoenix dactylifera*), sugarcane and pepper [5]. In recent years, with the continuous expansion of agricultural planting scale, the host range of phytoplasmas on Hainan Island has been expanding, and new phytoplasma strains and host plants have been continuously discovered [5,6,7]. For example, recent studies identified more than 12 new host plants of phytoplasmas on Hainan Island and proposed nine new 16Sr subgroups, enriching the understanding of phytoplasma diversity in the region [5].

The diversity of plant hosts and the phylogenetic evolution of phytoplasmas have significantly complicated epidemic monitoring as well as prevention and control of phytoplasma diseases on Hainan Island. The wide range of host plants makes it difficult to comprehensively monitor the occurrence and spread of phytoplasma diseases, as different hosts may exhibit different symptoms and have different transmission dynamics [9]. In addition, the phylogenetic evolution of phytoplasmas, including genetic mutations and horizontal gene transfer, may lead to changes in their pathogenicity, host adaptability, and transmission efficiency, making the existing detection methods and control strategies ineffective [9]. Therefore, a comprehensive review of the plant host diversity and phylogenetic evolution of phytoplasmas on Hainan Island is urgently needed, which can provide a theoretical basis for improving the accuracy of disease detection, optimizing epidemic monitoring systems, and formulating scientific and effective prevention and control strategies.

This review summarizes the research progress on the plant host diversity of phytoplasmas on Hainan Island, including the main types of phytoplasma diseases and their host plants. Phytoplasmas infecting different plant hosts, causing different symptoms, or belonging to different 16Sr subgroups were employed. Information about the phytoplasma strains and their 16S rRNA gene sequences were obtained from GenBank through the National Center for Biotechnology Information (https://www.ncbi.nlm.nih.gov/) on 27 March 2026. The review also focuses on the classification and phylogenetic evolution characteristics of phytoplasmas, the characteristics of mixed infection resulting from host diversity, and the diversity of transmission vectors. Furthermore, the challenges posed by host diversity and phylogenetic evolution to disease detection, monitoring, and prevention are discussed, and corresponding monitoring and control strategies are proposed. This review aims to provide a reference for the sustainable management of phytoplasma diseases on Hainan Island and the healthy development of tropical agriculture.

## 2. Diversity of Plant Hosts Infected by Phytoplasmas on Hainan Island

The diversity of plant hosts is one of the most prominent characteristics of phytoplasmas on Hainan Island, which is closely related to the rich tropical plant resources and unique ecological environment of the island [5,6,7]. Phytoplasmas on Hainan Island can infect a variety of plants, including tropical economic crops, ornamental plants, weeds, and wild plants, inducing different types of diseases according to the host species and phytoplasma strain, as listed in Table S1. Based on the typical symptoms induced by the phytoplasma infection, the phytoplasma diseases on Hainan Island can be divided into three main types, namely leaf-yellowing diseases, witches’ broom diseases, and floral malformation diseases.

### 2.1. Leaf-Yellowing Diseases

Leaf yellowing is one of the most common symptoms induced by phytoplasma infection, mainly manifested as chlorosis, yellowing, or bleaching of leaves, which gradually spreads from the leaf tip or edge to the entire leaf, eventually leading to leaf withering and abscission, and even plant death [5,6,7,8]. On Hainan Island, leaf-yellowing diseases caused by phytoplasmas mainly occur on important tropical economic crops, causing significant economic losses to agricultural production.

Areca palm yellow leaf disease (AYLD) is the most destructive phytoplasma disease on Hainan Island and has become a major threat to the areca palm industry [6,7,8]. Areca palm is an important tropical economic crop in Hainan Province. AYLD was first reported in Tunchang County, Hainan, in 1981, and has now spread to all cities and counties in Hainan. The disease can be caused by the phytoplasmas belonging to the 16SrI, 16SrII and 16SrXXXII groups [7,8,9]. The strains of ‘*Candidatus* Phytoplasma malaysianum’ belonging to the 16SrXXXII group and the strains from the 16SrII group have been found to be associated with AYLD, causing yellowing symptoms [7,8]. Recent studies have confirmed that the key pathogen of AYLD is ‘*Candidatus* Phytoplasma arecae’ belonging to the 16SrI group. A draft genome of this phytoplasma has been assembled, revealing its vertical transmission pathway through “diseased tree–seed–seedling” [9].

Sugarcane white leaf disease (SCWLD) is another important leaf-yellowing disease caused by phytoplasmas on Hainan Island [5]. Sugarcane is a major cash crop in Hainan, and SCWLD can cause significant yield losses and quality degradation. The disease is characterized by white or pale yellow stripes on the leaves, which gradually expand to the entire leaf, leading to leaf drying and plant stunting. Studies have shown that the phytoplasma causing SCWLD on Hainan Island belongs to the 16SrII group, which has a high homology with the peanut witches’ broom phytoplasma [5]. In recent years, with the expansion of sugarcane planting scale and the frequent circulation of seedlings, SCWLD has shown an increasing trend of occurrence on Hainan Island, posing a serious threat to the sugarcane industry.

Citrus huanglongbing (HLB) is a devastating disease of citrus cropsthat is mainly caused by *Candidatus* Liberibacter asiaticus (CLas). Recent studies have found that phytoplasmas can also be detected in citrus plants infected with CLas, forming a complex infection [7]. On Hainan Island, citrus is widely planted, and HLB is one of the main diseases affecting citrus production. The disease is characterized by mottled yellowing of leaves, uneven fruit ripening, and small, misshapen fruits, eventually leading to tree decline and death [7]. Studies have shown that ‘*Candidatus* Phytoplasma asteri’ (CPa)-related strains could be detected in citrus plants with HLB symptoms caused by CLas on Hainan Island [7]. The complex infection of CLas and CPa increases the difficulty of disease diagnosis and control, further threatening the citrus industry on Hainan Island.

### 2.2. Witches’ Broom Diseases

Witches’ broom is a typical symptom induced by phytoplasma infection, mainly manifested as excessive branching of the host plant, short internodes, small and deformed leaves, and the formation of a broom-like cluster of branches. This symptom is caused by the disruption of the host’s hormone balance by phytoplasmas, leading to abnormal growth of lateral buds [5]. On Hainan Island, witches’ broom diseases caused by phytoplasmas occur on a variety of plants, including food crops, economic crops, and weeds.

Peanut witches’ broom disease (PnWBD) is an important phytoplasma disease affecting peanut production on Hainan Island. Peanuts are an important oil and cash crop in Hainan, and PnWBD can cause significant yield losses. The disease is characterized by excessive branching of peanut plants, short internodes, small leaves, and failure to bear pods or small pods [5].

Chinaberry witches’ broom disease (CnWBD) is another common witches’ broom disease on Hainan Island, which occurs on chinaberry trees (*Melia azedarach*), an ornamental and afforestation tree in tropical and subtropical regions [5,10]. The disease is characterized by dense branching of the tree crown, small and yellow leaves, and stunted growth, affecting the ornamental value and growth of chinaberry trees. Although there are few systematic studies on CnWBD on Hainan Island, field investigations have shown that the disease is widely distributed on the island, and the phytoplasma involved belongs to the 16SrI group [5].

*Trema tomentosa* witches’ broom disease (TwBD) occurs on *Trema tomentosa*, a wild plant widely distributed on Hainan Island. The disease is caused by a phytoplasma belonging to the 16SrXXXII-D subgroup, which has 100% sequence similarity with the phytoplasma infecting areca palm on Hainan Island [11,12]. In addition, witches’ broom diseases caused by phytoplasmas have also been reported on other plants on Hainan Island, such as *Praxelis clematidea*, periwinkle and cassava [5].

### 2.3. Floral Malformation Diseases

Floral malformation is another important type of symptom induced by phytoplasma infection, mainly manifested as phyllody (floral organs transformed into leaf-like structures), virescence (floral organs turn green), and sterility, which can significantly affect the reproductive capacity of the host plant. On Hainan Island, floral malformation diseases caused by phytoplasmas mainly occur on ornamental plants and weeds, but some economic crops can also be infected [5].

Periwinkle phyllody disease is a typical floral malformation disease caused by phytoplasmas on Hainan Island. Periwinkle (*Catharanthus roseus*) is an ornamental plant in tropical regions. The phytoplasma causing periwinkle phyllody disease on Hainan Island is ‘*Candidatus* Phytoplasma asteri’ (CPa) [5]. Studies have shown that CPa alone can induce phyllody and internode shortening in periwinkle [5].

*Melochia corchorifolia* virescence disease is another floral malformation disease reported on Hainan Island. *Melochia corchorifolia* is a weed widely distributed in tropical and subtropical regions, and its virescence disease is characterized by the greening of floral organs, such as petals and sepals, and the transformation of floral buds into leaf-like structures [13]. It has been confirmed that the disease is caused by a phytoplasma, and the phytoplasma involved is speculated to belong to the 16SrI or the 16SrII group [13,14].

## 3. Systematic Classification of Phytoplasmas on the Island

**Figure 1:**
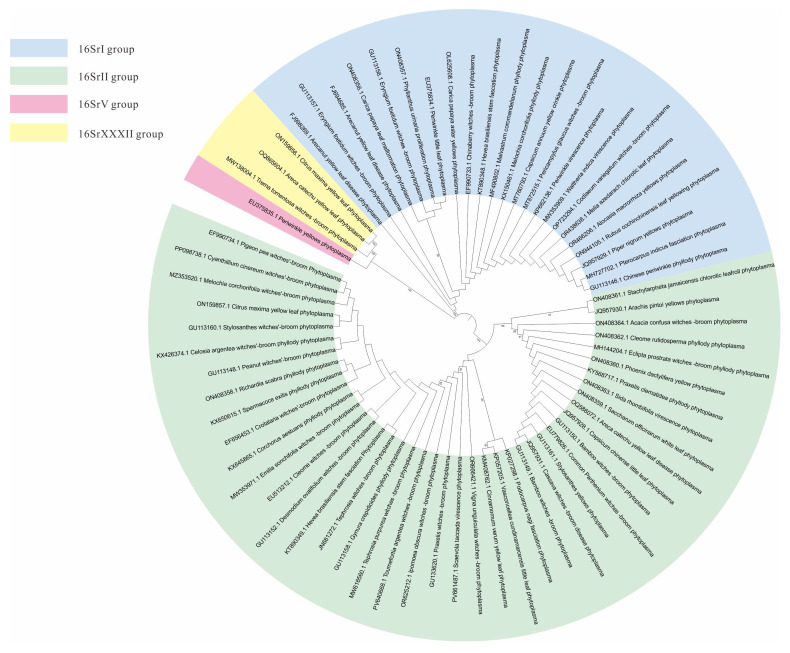
Phylogenetic tree based on the 16S rRNA gene sequences of the phytoplasmas identified on Hainan Island, China, and other selected phytoplasmas. The evolutionary history was constructed using the neighbor-joining method implemented in MEGA 11. Numbers on the branches represent bootstrap values from 1000 replicates (only values above 50 are shown). Detailed information on phytoplasma strains is provided in Table S1.

The systematic classification of phytoplasmas is mainly based on the sequence analysis of the 16S rRNA gene, which is a highly conserved gene in prokaryotes and can reflect the evolutionary relationship between different phytoplasma strains [2,3]. Based on restriction fragment length polymorphism (RFLP) analysis of 16S rRNA gene sequences, phytoplasmas are divided into different 16Sr groups and subgroups, which is the most widely used classification system, as shown in Figure 1. To date, based on RFLP analysis of 16S rRNA gene sequences employing the online tool *i*PhyClassifier (https://plantpathology.ba.ars.usda.gov/cgi-bin/resource/iphyclassifier.cgi, accessed on 7 June 2026), phytoplasmas on Hainan Island have been classified into four major 16Sr groups, namely 16SrI, 16SrII, 16SrV, and 16SrXXXII, with multiple subgroups, showing rich genetic diversity. Based on the reported records, as indicated in Table S1 and Figure 1, 16SrI and 16SrII are the most frequently reported groups, accounting for 33.33% and 60.87%, respectively, among the reported identified phytoplasma strains.

### 3.1. 16SrI Group Phytoplasmas

The 16SrI group (aster yellows group) is one of the most widely distributed and diverse phytoplasma groups in the world, which can infect a variety of plants and cause severe diseases [2,3]. On Hainan Island, 16SrI group phytoplasmas are mainly distributed in areca palm, chinaberry, periwinkle, and other plants, and are important pathogens causing leaf-yellowing and witches’ broom diseases [5,10,15,16,17,18,19,20,21,22,23,24,25,26,27,28,29,30].

A recent study has confirmed that the key pathogen of areca palm YLD on Hainan Island is ‘*Candidatus* Phytoplasma arecae’ of the 16SrI group, and its genome draft has been successfully assembled, providing a basis for studying its genetic characteristics and pathogenic mechanism [9]. In addition, a multi-locus sequence analysis (MLSA) of 16SrI group phytoplasmas in China has shown that there are significant genetic variations among different strains, and the strains from Hainan Island form an independent evolutionary branch [28]. A total of three new subgroups (16SrI-AP, 16SrI-AQ, and 16SrI-AR) have been proposed for the 16SrI group phytoplasmas on Hainan Island, enriching the classification system of this group [5].

### 3.2. 16SrII Group Phytoplasmas

The 16SrII group (peanut witches’ broom group) is another dominant phytoplasma group on Hainan Island, which is widely distributed in peanut, sugarcane, *Praxelis clematidea* and other plants [5]. This group of phytoplasmas has a high host adaptability and can infect both cultivated crops and weeds, playing an important role in the spread and evolution of phytoplasmas [5,31,32,33,33,34,35,36,37,38,39,40,41,42,43,44,45,46,47,48,49,50,51,52].

The 16SrII group phytoplasmas are the main pathogens causing peanut witches’ broom disease and sugarcane white leaf disease on Hainan Island [5,31,32,33,33,34,35,36,37,38,39,40,41,42,43,44,45,46,47,48,49,50,51,52]. A recent study proposed four new subgroups (16SrII-Y, 16SrII-Z, 16SrII-AB, and 16SrII-AC) for the 16SrII group phytoplasmas on Hainan Island, which are significantly different from the known subgroups in terms of genetic characteristics [5].

As shown in Figure 1, the 16SrII group phytoplasmas are the dominant group on Hainan Island. The discovery of new subgroups and new host plants indicates that the 16SrII group phytoplasmas on Hainan Island are still in the process of evolutionary adaptation, which may lead to the expansion of their host range and the aggravation of disease damage in the future.

### 3.3. 16SrV Group Phytoplasmas

The 16SrV group (elm yellows group) is a relatively less common phytoplasma group on Hainan Island, which is mainly distributed in wild plants and some ornamental plants [2]. This group of phytoplasmas is mainly characterized by inducing yellowing and witches’ broom symptoms in host plants and has a relatively narrow host range compared with the 16SrI and 16SrII groups [5,53,54,55,56,57,58].

Although there are few systematic studies on 16SrV group phytoplasmas on Hainan Island, field investigations and molecular detection have confirmed their presence in some plants, such as periwinkle with yellows disease [53]. The 16SrV group phytoplasmas on Hainan Island are speculated to be mainly transmitted by leafhoppers, and their genetic characteristics are significantly different from those in other regions of China, which may be related to the unique ecological environment of Hainan Island [20,53,54,55,56,57,58].

### 3.4. 16SrXXXII Group Phytoplasmas

The 16SrXXXII group is a relatively new phytoplasma group, which was first reported in Southeast Asia and has been gradually found in other regions in recent years [6,7,11,59,60,61,62,63]. On Hainan Island, 16SrXXXII group phytoplasma was first identified in *Trema tomentosa*, and the pathogens have since been reported in areca palm and *Citrus maxima*. The 16SrXXXII group phytoplasmas are important pathogens causing leaf-yellowing and witches’ broom diseases [6,7,11].

A recent study has found that the 16SrXXXII-D subgroup ‘*Candidatus* Phytoplasma malaysianum’ can infect areca palm on Hainan Island, causing yellowing symptoms, and this subgroup has been included in the international authoritative phytoplasma classification system *i*PhyClassifier [64]. In addition, new subgroups 16SrXXXII-D, 16SrXXXII-E, and 16SrXXXII-F have been proposed based on RFLP analysis, enriching the classification of the 16SrXXXII group phytoplasmas [6,7,11,63]. Based on current studies and their reported subgroup assignments, the 16SrXXXII-A, 16SrXXXII-B and 16SrXXXII-C subgroups are distributed in Southeast Asia (Malaysia), while the 16SrXXXII-D, 16SrXXXII-E and 16SrXXXII-F subgroups are only found in East Asia (Hainan, Yunnan, and Guangdong in China, as well as Japan and South Korea) [6,7,11,59,60,61,62,63].

## 4. Mixed Infection of Phytoplasmas on Hainan Island

The rich diversity of plant hosts on Hainan Island provides favorable conditions for the mixed infection of phytoplasmas. A plant could be infected by multiple phytoplasma strains. In addition, plants can be co-infected by phytoplasmas and other pathogens such as viruses, bacteria, and fungi [5,6,65]. Mixed infection of phytoplasmas and other pathogens on Hainan Island not only increases the complexity of disease symptoms but also affects the accuracy of disease diagnosis and the effectiveness of control strategies.

The simultaneous presence of multiple pathogens or strains in a single host has been found on Hainan Island. Areca palm plants on Hainan Island can be infected by 16SrI group *Candidatus* Phytoplasma arecae and 16SrXXXII-D subgroup ‘*Candidatus* Phytoplasma malaysianum’, leading to more severe yellowing symptoms and faster disease progression [6,8,9,66]. The phytoplasmas associated with AYLD detected in three Asian countries, China, India and Sri Lanka, belong to the 16SrI, 16SrXI, 16SrXIV and 16SrXXXII groups [6,8,9,67,68,69].

One 16Sr group or subgroup phytoplasma can infect multiple plant hosts [5]. The 16SrII group phytoplasma causing peanut witches’ broom disease can also infect peanut, sugarcane, *Praxelis clematidea* and other plants, forming a cross-host infection system [5]. The 16SrXXXII-D subgroup phytoplasma can infect areca palm, *Trema tomentosa* and *Citrus maxima*, showing a wide host adaptability [6,7]. This cross-host infection ability enables phytoplasmas to spread between different host plants, increasing the difficulty of epidemic monitoring and control.

The causal agents associated with disease symptoms including yellowing and leaf crinkling in *Citrus maxima* on Hainan Island were identified by Yu et al. [7]. In addition to ‘*Ca. L. asiaticus*’ present in symptomatic plants, two distinct phytoplasma strains, which belong to the 16SrII and 16SrXXXII groups, were also identified [7]. Among the 54 detected symptomatic samples, 22.2% tested positive for 16SrII phytoplasma, 3.7% for 16SrXXXII, and 11.1% for *Ca. L. asiaticus*. Furthermore, 7.4% of the samples exhibited mixed infections involving both phytoplasma and *Ca. L. asiaticus* [7]. Lin et al. found that areca palms showing yellow leaf symptoms can be infected by areca palm yellow leaf phytoplasma (AYLP) belonging to the 16SrI-B subgroup and areca palm velarivirus 1 (APV1). Among 520 samples tested by qPCR, AYLP was detected in 23.46% (n = 122), APV1 in 53.46% (n = 278), and a co-infection with both AYLP and APV1 in 10.96% (n = 57) [66].

Under natural conditions, plants can be simultaneously infected by phytoplasmas belonging to different 16Sr groups or by phytoplasmas along with other pathogens such as ‘*Candidatus* Liberibacter’ species, viruses, spiroplasmas, fungi, and other difficult-to-culture phloem-limited bacteria [70,71,72,73,74,75,76,77,78,79,80,81,82,83,84,85,86]. The mixed infection of multiple pathogens can lead to the superposition of symptoms, making it difficult to distinguish the pathogenic factors and also reducing the effectiveness of single control measures.

## 5. Diversity of Phytoplasma Transmission Routes and Reservoirs

The diversity of transmission routes, insect vectors, and reservoir hosts is an important factor affecting the spread and epidemic of phytoplasma diseases. On Hainan Island, the diversity of plant hosts and the suitable climatic environment have led to the diversity of phytoplasma transmission routes including plant seeds and seedlings, insect vectors, and reservoir hosts [9].

### 5.1. Seeds and Seedlings

The transmission of phytoplasmas via seeds and seedlings is an important long-distance transmission pathway that is closely related to the circulation of plant germplasm resources [86]. On Hainan Island, the frequent circulation of seeds and seedlings of tropical economic crops, such as areca palm, has become an important means for the long-distance spread of phytoplasmas [6,9]. Recent studies have found that areca palm YLD phytoplasma can be vertically transmitted through “diseased tree–seed–seedling”, which is the first report of vertical transmission of areca palm YLD phytoplasma [9,86]. This finding indicates that the seeds and seedlings of diseased areca palm can carry phytoplasmas, and the long-distance transportation of these seeds and seedlings can lead to the spread of the disease to new regions.

The transmission of YLD phytoplasmas via seeds and seedlings of areca palm poses a serious threat to the quarantine of plant germplasm resources. The lack of effective detection methods for YLD phytoplasmas in seeds and seedlings may lead to the introduction of new phytoplasma strains and the expansion of disease distribution. Therefore, strengthening the detection and quarantine of areca palm seeds and seedlings is an important measure to prevent the long-distance spread of YLD phytoplasma and their related diseases.

### 5.2. Insect Vectors

Insects are the main transmission carriers of phytoplasmas, and most phytoplasmas are transmitted by phloem-feeding insects, such as leafhoppers, planthoppers and aphids [86,87,88,89,90]. On Hainan Island, the warm and humid climate is suitable for the survival and reproduction of vector insects, leading to a high diversity of vector insects and strong transmission capacity [9].

Piercing–sucking insects are important vector insects of phytoplasmas on Hainan Island. For example, piercing–sucking insects might be the vector insects of areca palm YLD phytoplasma, which can transmit the phytoplasma between areca palm plants through feeding [9,86]. The occurrence of these vector insects is closely related to the planting environment of areca palm; high planting density and poor ventilation can increase the population density of vector insects, thereby promoting disease spread.

The diversity of insect vectors and their strong adaptability make the transmission of phytoplasmas on Hainan Island very complex. Different vector insects have different host ranges and transmission efficiencies, which affects the spread speed and epidemic scope of phytoplasma diseases [86]. In addition, the resistance of vector insects to insecticides also increases the difficulty of controlling the transmission of phytoplasmas. Based on the current situation, more accurate information about insect vectors of phytoplasma on Hainan Island needs to be obtained via in-depth research and reported.

### 5.3. Reservoir Hosts

Reservoir hosts refer to plants that can carry phytoplasmas, which play an important role in their spread and survival [5,6,7]. The rich diversity of wild plants and weeds provides a large number of reservoir hosts for phytoplasmas, which are important “reservoirs” of phytoplasmas [5,6,7,65].

Recent studies have found that chinaberry, *Trema tomentosa*, papaya, and pomelo may be potential reservoir hosts of areca palm yellow leaf phytoplasma [5,6,7]. Among them, the 16S rRNA gene sequence of the phytoplasma in chinaberry and papaya has a similarity of 100% with that of the areca palm yellow leaf phytoplasma, indicating that these plants can carry the phytoplasma and may transmit it to areca palm through vector insects [5,6,7,9]. In addition, weeds such as *Melochia corchorifolia* and *Waltheria indica* can also serve as reservoir hosts of 16SrII group phytoplasmas, carrying related phytoplasmas [5].

*Praxelis clematidea*, an invasive weed on Hainan Island, is an important intermediate host of 16SrII group phytoplasmas [5,20]. Although *Praxelis clematidea* can show witches’ broom symptoms after being infected by phytoplasmas, it can also survive for a long time and serve as a source of infection for other plants [20]. In addition, wild plants such as *Trema tomentosa* and *Waltheria indica* can also serve as reservoir hosts of phytoplasmas, carrying phytoplasmas and transmitting them to economic crops through vector insects [20].

The existence of reservoir hosts increases the difficulty of epidemic monitoring and control of phytoplasma diseases. Since reservoir hosts do not show obvious symptoms, it is difficult to detect and remove them, leading to the long-term survival and spread of phytoplasmas [5,6,7,20]. Therefore, identifying and monitoring the reservoir hosts of phytoplasmas is an important part of the comprehensive prevention and control of phytoplasma diseases on Hainan Island.

## 6. Challenges to Epidemic Monitoring and Prevention of Related Diseases

The diversity of plant hosts and the phylogenetic evolution of phytoplasmas on Hainan Island have brought great challenges to the detection, epidemic monitoring, and prevention of phytoplasma diseases. These challenges are mainly reflected in the complexity of disease diagnosis, the difficulty of epidemic monitoring, and the ineffectiveness of single control measures [20]. Based on these challenges, corresponding monitoring and prevention strategies are proposed to provide a basis for the sustainable management of phytoplasma diseases on Hainan Island.

Host diversity and phylogenetic evolution have led to complexity of disease detection and diagnosis of phytoplasma diseases. The wide host range of phytoplasmas on Hainan Island leads to diverse disease symptoms, and different hosts infected by the same phytoplasma strain may show different symptoms, while the same host infected by different phytoplasma strains may show similar symptoms [5,6,7]. For example, yellowing symptoms can be caused by the 16SrI group, the 16SrXXXII group, and other phytoplasma strains, making it difficult to distinguish the pathogenic strains by symptoms alone [6,16,17]. In addition, the phylogenetic evolution of phytoplasmas may lead to changes in their genetic characteristics, making the existing detection methods (such as PCR) ineffective, further increasing the difficulty of disease diagnosis [20]. For palm crops such as areca palm and coconut, the difficulty of phytoplasma detection is further increased due to their own characteristics.

Host diversity and phylogenetic evolution have also led to difficulty in epidemic monitoring of phytoplasma diseases. The rich diversity of plant hosts and transmission vectors makes the spread of phytoplasma diseases very complex [65,66]. Reservoir hosts and asymptomatic infected plants can serve as sources of infection for a long time, making it difficult to accurately grasp the epidemic scope and spread trend of the disease [5,20]. In addition, the frequent circulation of seeds and seedlings and the movement of vector insects further promote the spread of phytoplasmas, making epidemic monitoring more difficult [65]. For palm crops, the existence of reservoir hosts brings great difficulties to the monitoring and early warning of phytoplasma diseases.

Host diversity and phylogenetic evolution have also resulted in complexity of prevention and control of phytoplasma diseases. Co-infection of phytoplasmas, coupled with the diversity of transmission vectors and reservoir hosts, makes single control measures (such as chemical control or biological control) ineffective. For palm crops, the problems of host diversity, reservoir hosts, and complex infection make it impossible to use a single method in the process of prevention and control, and multiple methods must be used together for comprehensive prevention and control [20]. Furthermore, the phylogenetic evolution of phytoplasmas may lead to the loss of resistance of disease-resistant varieties, further increasing the difficulty of prevention and control. The unique geographical environment of Hainan Island (such as the intercropping of different crops) also increases the complexity of prevention and control measures.

Another issue caused by host diversity and phylogenetic evolution is the difficulty in breeding disease-resistant varieties of the phytoplasma diseases. Considering the problems of host diversity and complex infection, it is necessary to take these factors into account in the breeding of disease-resistant varieties to prevent the loss of disease resistance caused by these problems. For palm crops, the diversity of phytoplasma strains and the occurrence of complex infection make it more difficult to breed disease-resistant varieties, which requires screening varieties for comprehensive resistance to multiple phytoplasma strains.

## 7. Prevention and Control Strategies

Strengthening the detection and quarantine of phytoplasmas is important to preventing the spread of phytoplasma diseases. For port quarantine, it is necessary to expand the scope of detection from seeds and seedlings to reservoir host plants that may carry phytoplasmas, so as to prevent the introduction of new phytoplasma strains [6,28]. At the same time, it is necessary to improve the detection technology of phytoplasmas, such as nested PCR, LAMP and droplet digital PCR [91,92]. Rapid and visual method LAMP and absolute quantification droplet digital PCR are more suitable for the detection of phytoplasmas on Hainan Island. It is necessary to establish a regular detection system for phytoplasma diseases in key planting areas, timely find and eliminate infected plants, and cut off the source of infection.

Due to the complexity of phytoplasma diseases on Hainan Island, it is important to adopt comprehensive prevention and control measures combining agricultural control, chemical control, and biological control. For agricultural control, it is necessary to strengthen the management of planting density, improve ventilation and light conditions, and reduce the population density of insect vectors [5,6,7].

Breeding disease-resistant varieties is the most economical and effective measure for the long-term control of phytoplasma diseases. In the process of breeding disease-resistant varieties, it is necessary to fully consider the diversity of phytoplasma strains and the problem of complex infection, so as to avoid the loss of resistance caused by the phylogenetic evolution of phytoplasmas [93,94]. As a suggestion, in the breeding of disease-resistant areca palm varieties, it is necessary to screen varieties resistant to both 16SrI group and 16SrXXXII group phytoplasmas, so as to improve the comprehensive resistance of varieties [6,9]. In addition, it is useful to develop molecular-marker-assisted breeding technology to accelerate the breeding process of disease-resistant varieties and improve the breeding efficiency.

## 8. Conclusions and Future Perspectives

Hainan Island, with its unique tropical ecological environment, has a rich diversity of phytoplasmas and plant hosts. Currently, comparatively more reports have been made on the aspects of identification of phytoplasma diseases on the island, providing relatively abundant insights into the occurrence of phytoplasma diseases.

However, aiming to prevent phytoplasma diseases on Hainan Island, there is little or almost no information about insect vector identification and their transmission experiments, disease-resistant breeding, or genome sequencing. Therefore, it is necessary to strengthen the research aspects such as insect vector identification and their transmission experiments, disease-resistant breeding, genome sequencing, multilocus typing, long-term surveillance, and standardized diagnostics in the future.

In brief, the plant host diversity and phylogenetic evolution of phytoplasmas on Hainan Island are important research topics in tropical plant pathology. In-depth study of these issues can not only enrich the understanding of phytoplasma diversity and evolution but also provide a theoretical basis for the scientific prevention and control of phytoplasma diseases, promoting the healthy development of tropical agriculture on the island.

## Data Availability

No new data were created or analyzed in this study. Data sharing is not applicable to this article.

## Supplementary Materials

The following supporting information can be downloaded at: https://www.mdpi.com/article/10.3390/plants15121787/s1, Table S1. Information of the represent phytoplasmas identified in Hainan Island of China..
