# Supplementary material for: Host Diversity and Phylogenetic Evolution of Phytoplasmas on Hainan Island in China, Bringing Challenges to Monitoring and Prevention of Related Plant Diseases
Source: Plants (Basel). 2026 Jun 10;15(12):1787. doi: 10.3390/plants15121787 (PMC13306697; doi:10.3390/plants15121787)
Supplement: Supplementary file 1 [file plants-15-01787-s001.zip › 260605 plants-4341900-Table S1.pdf]

Table S1. Information of the represent phytoplasmas identified in Hainan Island of China.

No.	Phytoplasma strains	Identifier	Host	Disease symptoms	Sampling location	Sampling years	16Sr groups	Accession No. (16S rRNA)	References
1	*Alocasia macrorrhiza* yellows	AmY-hn	*Alocasia macrorrhiza*	chlorotic ovoid leaves that turned yellow from green gradually, yellowing of mesophyll tissue, miniature leaves, and systemic wilting.	Qionghai, Hainan	2022	16SrI-B	OR466206	[15]
2	Arecanut yellow leaf	AYL-B165	*Areca cathecu*	Yellow leaf	Qionghai, Hainan	2008-2010	16SrI-L	FJ694685	[16]
3	Arecanut yellow leaf	AYL-Wanning	*Areca cathecu*	Yellow leaf	Wanning, Hainan	2008-2009	16SrI-B	FJ998269	[17]
4	*Carica papaya* leaf malformation	CaPLM-HK1	*Carica papaya*	Leaf malformation	Haikou, Hainan	2019	16SrI-AP	ON408356	[5]
5	*Carica papaya* yellows	CpY-hnwn	*Carica papaya*	Yellows	Wanning, Hainan	2021	16SrI-B	OL625608	[18]
6	Chinaberry witches'-broom	CWB-Hn	*Melia azedarach*	Witches'-broom	Danzhou, Hainan	2007	16Sr I-B	EF990733	[19]
7	*Codiaeum variegatum' witches'-broom phytoplasma clone BYM*	BYM	*Codiaeum variegatum*	Witches'-broom	Qionghai, Hainan	2022	16SrI	OP723294	[20]
8	*Eryngium foetidum* witches'-broom	ErFWB1	*Eryngium foetidum*#	Witches'-broom	Danzhou, Hainan	2009	16SrI-B	GU113156	[5]
9	*Eryngium foetidum* witches'-broom	ErFWB3	*Eryngium foetidum*#	Witches'-broom	Danzhou, Hainan	2009	16SrI-AQ	GU113157	[5]

10	*Hevea brasiliensis* stem fasciation	RTSF-DS1A	*Hevea brasiliensis*	Stem fasciation	Danzhou, Hainan	2012-2013	16SrI-B	KT890348	[21]
11	*Malvastrum coromandelianum* phyllody	MCP-Chengmai 01	*Malvastrum coromandelianum*	Phyllody	Chengmai, Hainan	2017	16SrI-B	MF490802	[22]
12	*'Melia azedarach' chlorotic leaf phytoplasma isolate MaCL-hn*	MaCL-hn	*Melia azedarach*	Chlorotic Leaf	Wanning, Hainan	2021	16SrI-B	OR438638	[23]
13	*Melochia corchorifolia* phyllody	MCp1	*Melochia corchorifolia*	Phyllody	Haikou, Hainan	2015	16SrI-B	KX150461	[13]
14	*Rubus cochinchinensis' leaf yellowing phytoplasma clone HN1*	RcT-HN1	*Rubus cochinchinensis*	Chlorosis spread along the direction of vascular tissue while the leaf veinsremained green	Tunchang, Hainan	2022	16SrI-B	ON944105	[23]
15	Pepper yellow crinkle	PYC-hnhk	*Capsicum annuum*	Yellow crinkle	Haikou, Hainan	2020	16SrI-B	MT760793	[24]
16	*Pericampylus glaucus* witches'-broom	PgWB-hnda	*Pericampylus glaucus*	Witches'-broom	Ding' an, Hainan	2020	16SrI-B	MT872515	[25]
17	Periwinkle little leaf	PLL-Hn	*Catharanthus roseus*	Little leaf	Danzhou,, Hainan	2006	16SrI-B	EU375834	[26]
18	Periwinkle phyllody	PP-Hn2	*Catharanthus roseus*	Phyllody, inflorescences become clustered, petals gradually turn green, flowers wither and drop	Danzhou,, Hainan	2009	16SrI-B	GU113146	[27]

19	Periwinkle virescence	PeV-hnhk	*Catharanthus roseus*	Virescence	Haikou, Hainan	2013-2014	16SrI-B	KP662136	[28]
20	*Phyllanthus urinaria* proliferation	PhUP-HK2	*Phyllanthus urinaria*	Proliferation	Haikou, Hainan	2019	16SrI-AR	ON408357	[5]
21	*Piper nigrum* yellow	PepY-alp2	*Piper nigrum*#	Yellow	Wanning, Hainan	2011-2016	16Sr I-B	JQ957929	[5]
22	*Pterocarpus indicus* fasciation	PtIFP-Haikou	*Pterocarpus indicus*	Fasciation	Haikou, Hainan	2018	16SrI-AS	MH727702	[29]
23	*Waltheria indica* virescence	WiV-hnda	*Waltheria indica*	Floral virescence, leaf chlorosis, and leaflet	Ding'an, Hainan	2020	16SrI-B	MW353909	[30]
24	*Acacia confusa* witches'-broom	AcCWB-HK6	*Acacia confusa*#	Witches'-broom	Haikou, Hainan	2020	16SrII-A	ON408364	[5]
25	*Arachis pintoi* yellow	APY-alp3	*Arachis pintoi*#	Yellow	Danzhou, Hainan	2010	16SrII-Y	JQ957930	[5]
26	Areca catechu' yellow leaf disease phytoplasma	AYL-HNWC5	*Areca cathecu*	Yellow leaf	Wenchang, Hainan	2022	16SrII	OQ586072	[8]
27	Bamboo witches'-broom	BWB-Hn1	*Dendrocalamus giganteus*#	Witches'-broom	Wanning, Hainan	2009	16SrII-Z	GU113149	[5]
28	Bamboo witches'-broom	BWB-Hn2	*Dendrocalamus giganteus*	Witches'-broom	Qionghai, Hainan	2009	16SrII-A	GU113150	[5]
29	*Capsicum chinense* little leaf	CCLL-alp1	*Capsicum chinense*	Little leaf, Distorted leaf veins	Danzhou, Hainan	2011	16SrII-A	JQ957928	[31]

30	Cassava witches'-broom	CasWB-alp4	*Manihot esculenta*[#]	Witches'-broom	Wanning, Hainan	2010	16SrII-A	JQ957931	[5]
31	*Celosia argentea* witches'-broom phyllody	Ca1	*Celosia argentea*	Witches'-broom and phyllody	Haikou, Hainan	2015	16SrII-A	KX426374	[32]
32	*Citrus maxima* yellow leaf	CmPII-hn	*Citrus maxima*	Yellowing and mottled leaves	Ding'an, Hainan	2020-2021	16SrII-V	ON159857	[7]
33	*Cinnamomum verum* yellow leaf	CYL	*Cinnamomum verum*	Yellow leaf	Wanning, Hainan	2013	16SrII-A	KM408762	[33]
34	*Cleome rutidosperma* phyllody	ClRP-HK5	*Cleome rutidosperma*	Phyllody	Haikou, Hainan	2021	16SrII-A	ON408362	[5]
35	*Cleome* witches'-broom	ClWB-Hnsy	*Cleome viscosa*	Witches'-broom	Sanya, Hainan	2004	16SrII-A	EU513212	[34]
36	*Parthenium hysterophorus* witches'-broom	PHWB-YJJ2	*Parthenium hysterophorus*	Witches'-broom	Danzhou, Hainan	2008	16SrII-A	EU779826	[35]
37	*Corchorus aestuans* phyllody	CAP-P1P7HnHk	*Corchorus aestuans*	Phyllody, red leaves	Haikou, Hainan	2015	16SrII-A	KX645865	[36]
38	*Crotalaria* witches'-broom	CrWB-ef3	*Crotalaria sessiliflora*	Witches'-broom	Danzhou, Hainan	2006	16SrII-A	EF656453	[37]
39	*Cyanthillium cinereum' witches'-broom phytoplasma clone CcWB-hnda*	CcWB-hnda	*Cyanthillium cinereum*	witches' broom	Ding'an, Hainan	2020	16SrII-A	PP098738	[38]

40	*Desmodium ovalifolium* witches'-broom	DeOWB	*Desmodium ovalifolium*[#]	Witches'-broom	Baisha, Hainan	2009	16Sr II-A	GU113152	[5]
41	*Eclipta prostrata* witches'-broom phyllody	Ep1	*Eclipta prostrata*	Witches'-broom and phyllody	Haikou, Hainan	2015	16SrII-A	MH144204	[39]
42	*Emilia sonchifolia* witches'-broom	EsWB-hnda	*Emilia sonchifolia*	Witches'-broom, internode shortening, leaf chlorosis,and leaflet	Hainan	2020	16SrII-V	MW353971	[40]
43	*Gynura crepidioides* phyllody	GP1	*Gynura crepidioides*	Clustered leaf buds, reduced leaf size, clustered inflorescences, and virescence of floral organs	Qionghai, Hainan	2006	16SrII-A	GU113158	[41]
44	*Hevea brasiliensis* stem fasciation	RTSF-DS1B	*Hevea brasiliensis*	Stem fasciation, irregular bending of branches, witches'-broom, little leaves on abnormal branches, and early withering of abnormal branches	Danzhou, Hainan	2012-2013	16SrII-A	KT890349	[21]
45	*Ipomoea obscura witches'-broom phytoplasma isolate IoWB-hnld*	IoWB-hnld	Ipomoea obscura	Witches'-broom, internode shortening, and small leaves	Ledong, Hainan	2020-2021	16SrII-A	OR625212	[42]

46	*Melochia corchorifolia* witches'-broom	McWB-hnda	*Melochia corchorifolia*	Witches -broom, leaf chlorosis, and leafletand internode shortening	Ding'an, Hainan	2020	16SrII-V	MZ353520	[14]
47	Papaya little leaf	PaLL-DZ01	*Carica papaya*	Little leaves, foliar chlorosis and axillary shoot proliferation	Danzhou, Hainan	2012-2014	16SrII-U	KP057205	[43]
48	Peanut witches'-broom	PnWB-Hn1	*Arachis hypogaea*	Witches'-broom	Dongfang, Hainan	2009	16SrII-A	GU113148	[5]
49	*Phoenix dactylifera* yellow	PhDY-HK3	*Phoenix dactylifera*#	Yellow	Haikou, Hainan	2022	16SrII-A	ON408360	[5]
50	Pigeon pea witches'-broom	PPWB-Hn	*Cajanus cajan*	witches'-broom	Danzhou, Hainan	2008	16SrII-A	EF990734	[44]
51	*Podocarpus nagi* fasciation	PNSF-TransT1	*Podocarpus nagi*	Fasciation	Wanning, Hainan	2014	16SrII-U	KP027298	[45]
52	*Praxelis clematidea* phyllody	PCP-CM01	*Praxelis clematidea*	Phyllody and witches'-broom	Chengmai, Hainan	2016	16SrII-V	KY568717	[46]
53	*Praxelis clematidea* witches'-broom	PrWB-Hn	*Praxelis clematidea*	Witches'-broom	Ledong, Hainan	2009	16Sr II-A	GU133620	[5]
54	*Richardia scabra* phyllody	RiSP-WZS	*Richardia scabra*#	Phyllody	Wuzhishan, Hainan	2021	16SrII-A	ON408358	[5]
55	*Scaevola taccada' virescence phytoplasma*	StV-hnss	*Scaevola taccada*	Virescence and small leaf	Sansha, Hainan	2023	16SrII	PV661487	[46]

56	*Sida rhombifolia* virescence	SiRV-LS	*Sida rhombifolia*#	Virescence	Lingshui, Hainan	2021	16SrII-A	ON408363	[5]
57	*Spermacoce exilis* phyllody	BRP-HnHk	*Spermacoce exilis*	Phyllody	Haikou, Hainan	2015	16SrII-AC	KX650615	[47]
58	*Stachytarpheta jamaicensis* chlorotic leafroll	StJCL-HK4	*Stachytarpheta jamaicensis*#	Chlorotic leafroll	Haikou, Hainan	2021	16SrII-AB	ON408361	[5]
59	*Stylosanthes* witches'-broom	StyWB	*Stylosanthes guianensis*	Witches'-broom, little leaf and plant stunting	Danzhou, Hainan	2008	16SrII-A	GU113160	[48]
60	*Stylosanthes* yellows	StyY	*Stylosanthes guianensis*	yellows	Danzhou, Hainan	2008	16SrII-A	GU113161	[48]
61	Sugarcane white leaf	SCWL-DZCM	*Saccharum officinarum*#	white leaf	Chengmai, Hainan	2021	16SrII-A	ON408359	[5]
62	*Tephrosia purpurea* witches'-broom	TeWB-Hnsy2005	*Tephrosia purpurea*	Witches'-broom	Sanya, Hainan	2005	16SrII-A	JN681272	[49]
63	*Tephrosia purpurea* witches'-broom	TpWB-hnld	*Tephrosia purpurea*	Witches'-broom, intemnode shortening, leaf chlorosis and leaflet formation	Ledong, Hainan	2021	16SrII-V	MW616560	[50]
64	*Tournefortia argentea' witches'-broom phytoplasma clone TaWB*	TaWB	*Tournefortia argentea*	Witches' broom, little leaf, internode shortening, and phyllody	Sansha, Hainan	2025	16SrII	PV640668	[51]
65	*Vigna unguiculata' witches'-broom phytoplasma clone HNNKY-3*		*Vigna unguiculata*	Reduced leaf size, chlorosis, and broom-like branch deformities	Haikou, Hainan	2023	16SrII	OR666421	[52]

66	Periwinkle yellows	PY-Hn	*Catharanthus roseus*	Yellows, clustered leaves, reduced leaf size	Danzhou, Hainan	2007	16SrV-F	EU375835	[53]
67	*Trema tomentosa* witches'-broom	TtWB-hn	*Trema tomentosa*	Witches'-broom, internodeshortening, and leaf chlorosis	Ding'an and Qionghai, Hainan	2020	16SrXXXII-D	MW138004	[11]
68	*Citrus maxima* yellow leaf	CmPXXXII-hn	*Citrus maxima*	Yellowing and mottled leaves	Ding'an, Hainan	2020-2021	16SrXXXII-D	ON159856	[7]
69	*'Areca catechu' yellow leaf phytoplasma clone AcY-hn1*	AcY-hn1	*Areca cathecu*	Yellow leaf	Ding'an, Hainan	2022	16SrXXXII	OQ865604	[6]
